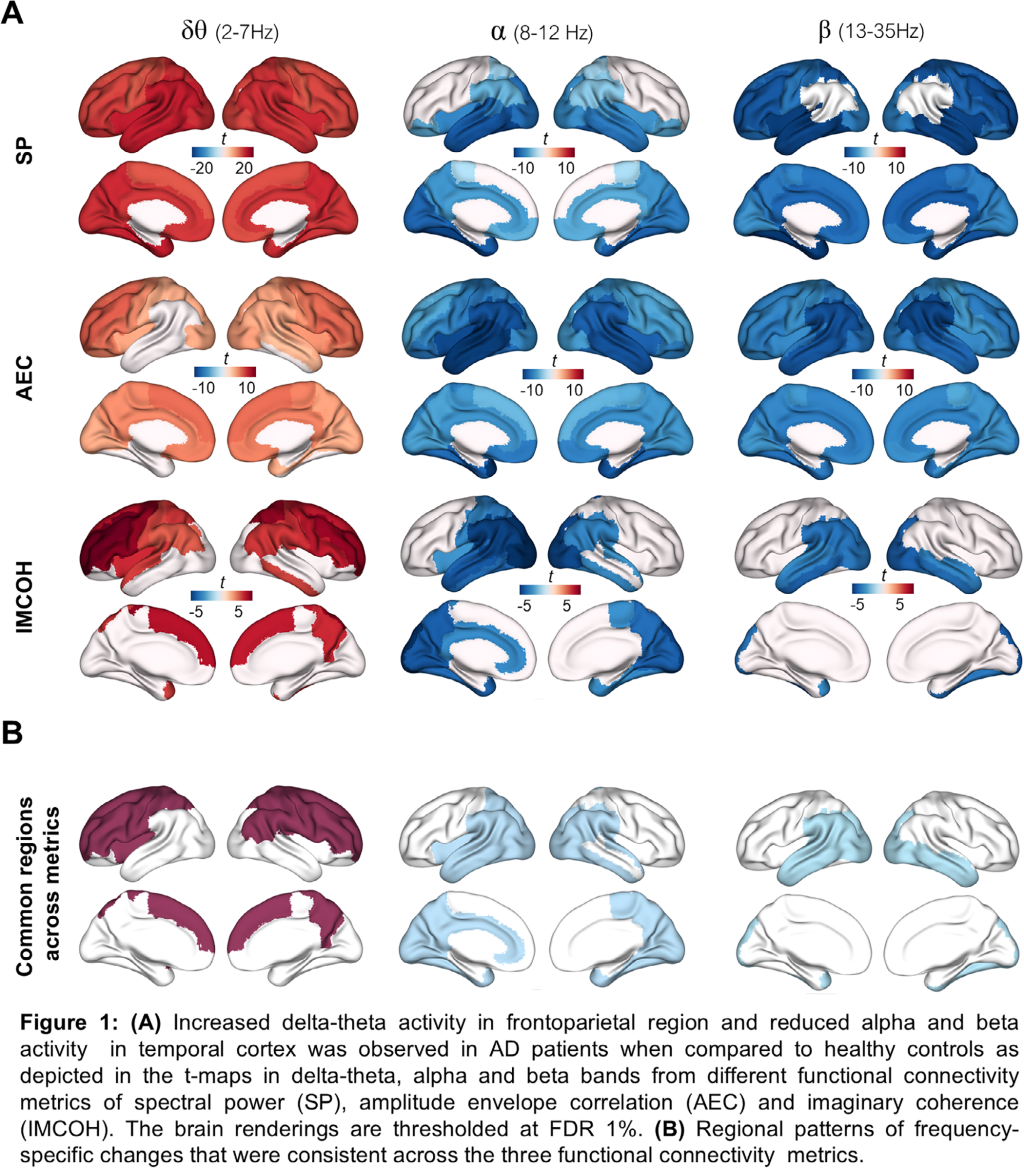# Regional Functional Connectivity in Alzheimer's and Controls: MEG Analysis Using Multiple Measures

**DOI:** 10.1002/alz70856_098440

**Published:** 2025-12-24

**Authors:** Pooja Prabhu, Kamalini G Ranasinghe, Kiwamu Kudo, Leighton B Hinkley, Anne Findlay, Bruce L. Miller, Joel H Kramer, Katherine P Rankin, Paul Garcia, Heidi Kirsch, John F Houde, Keith Vossel, Srikantan S Nagarajan

**Affiliations:** ^1^ University of California San Francisco, San Francisco, CA, USA; ^2^ Medical Imaging Business Center, Ricoh Company, Kanazawa, Japan; ^3^ UCSF, San Francisco, CA, USA; ^4^ Memory and Aging Center, Weill Institute for Neurosciences, University of California, San Francisco, San Francisco, CA, USA; ^5^ Memory and Aging Center, UCSF Weill Institute for Neurosciences, University of California, San Francisco, San Francisco, CA, USA; ^6^ Memory and Aging Center, Weill Institute for Neurosciences, University of California San Francisco, San Francisco, CA, USA; ^7^ University of California Los Angeles, Los Angeles, CA, USA; ^8^ Department of Radiology and Biomedical Imaging, University of California, San Francisco, San Francisco, CA, USA

## Abstract

**Background:**

Changes in brain network function have been clearly demonstrated in patients with Alzheimer's disease (AD). Specifically, previous electrophysiological studies have shown that delta and theta oscillatory activity increases in AD, while alpha and beta activity reduces, compared to controls. These frequency‐specific abnormalities have also shown to be region dependent, where low frequency delta‐theta increases are more predominant in the frontal cortices while alpha and beta reductions are more predominant in the temporal and parietal cortices, in AD. What is unknown is how consistent these frequency‐specific and region‐dependent abnormalities are, across different metrics of oscillatory activity ranging from local to long‐range connectivity.

**Method:**

Here, in a well characterized, AD biomarker positive cohort of 77 AD patients and age‐matched controls (*n* = 90), we used magnetoencephalography (MEG) to examine the local and long‐range oscillatory abnormalities. Specifically, source‐space reconstructed MEG signal for 40 cortical regions of Brainnetome was used to compute three different metric: local neural synchrony estimated as regional spectral power (SP); long‐range synchrony at slow time‐scale estimated from amplitude‐envelope correlation (AEC); and long‐range synchrony at fast time‐scale estimated from imaginary coherence (IMCOH). Each measure was computed for 2–7 Hz (delta‐theta), 8–12 Hz (alpha), and 15–29 Hz (beta) bands.

**Result:**

Consistent with previous results, we found that increased delta‐theta and reduced alpha and beta oscillatory activity patterns in AD compared to controls (Figure 1A). A conjoint analysis, in which we examined the common spatial patterns across different metrics of connectivity demonstrated that the frequency‐specific patterns have consistent regional dependencies (Fig‐1B). For example, the highest activity increases in delta‐theta was found in the dorsal frontal and anterior cingulate cortices, while the greatest reductions in alpha was consistently found in the inferolateral temporal cortices and posterior temporoparietal regions. Reductions in beta also showed regional consistencies like alpha.

**Conclusion:**

Our results show that frequency‐specific, region‐dependent neurophysiological manifestations in AD are conserved across different synchronization paradigms that contribute to the functional architecture of neural networks. Importantly, the current findings define unified region‐of‐interests within each frequency component (delta‐theta, alpha and beta) that can be further interrogated in future studies to investigate other pathobiological relationships in AD.